# Metatranscriptomic Sequencing of Winter and Spring Planktonic Communities from Lake Erie, a Laurentian Great Lake

**DOI:** 10.1128/mra.00351-22

**Published:** 2022-06-02

**Authors:** Brittany N. Zepernick, Elizabeth R. Denison, Justin D. Chaffin, George S. Bullerjahn, Christa P. Pennacchio, Thijs Frenken, Daniel H. Peck, James T. Anderson, Derek Niles, Arthur Zastepa, R. Michael L. McKay, Steven W. Wilhelm

**Affiliations:** a Department of Microbiology, University of Tennessee, Knoxville, Tennessee, USA; b Stone Laboratory and Ohio Sea Grant, Ohio State University, Put-in-Bay, Ohio, USA; c Department of Biological Sciences, Bowling Green State University, Bowling Green, Ohio, USA; d Department of Energy Joint Genome Institute, Lawrence Berkeley National Laboratory, Berkeley, California, USA; e Great Lakes Institute for Environmental Research, University of Windsor, Windsor, Ontario, Canada; f U.S. Coast Guard Cutter Neah Bay (WTGB 105), United States Coast Guard 9th District, Cleveland, Ohio, USA; g Orange Force Marine Ltd., Port Stanley, Ontario, Canada; h Environment and Climate Change Canada, Canada Centre for Inland Waters, Burlington, Ontario, Canada; Vanderbilt University

## Abstract

Previous reports suggest planktonic and under-ice winter microbial communities in Lake Erie are dominated by diatoms. Here, we report the assembled metatranscriptomes of 79 Lake Erie surface water microbial communities spanning both the winter (28 samples) and spring (51 samples) months over spatial, temporal, and climatic gradients in 2019 through 2020.

## ANNOUNCEMENT

Lake Erie’s winter phytoplankton blooms have been documented for decades (1), and recent studies have revealed that these communities are dominated by centric, colonial diatoms, including Aulacoseira islandica and Stephanodiscus binderanus (2–4). However, the ecophysiology of these blooms and comprehensive analyses of the winter community have received limited attention (5). Here, we report 28 winter and 51 spring Lake Erie metatranscriptomes collected across spatial, temporal, and climatic gradients in an effort to address these knowledge gaps.

Opportunistic samples were collected by U.S. Coast Guard Cutter *Neah Bay* between February and March in 2019 and 2020 (6). Additional spring samples were collected in May and June of these same years by Canadian Coast Guard Ship (CCGS) *Limnos* and M/V *Orange Apex*, respectively. Sampling occurred in both the western and central basins of Lake Erie. Water column parameters were recorded prior to each sampling, along with meteorological conditions and ice cover. The samples were additionally analyzed for nutrient concentrations and phytoplankton taxonomy (7). Water column samples and plankton net concentrated samples were immediately processed onboard ship. Briefly, the samples were filtered through 0.22-μm nominal pore-size filters, flash-frozen, and stored at −80°C until extraction. RNA was extracted using standard phenol-chloroform methods with ethanol precipitation (8). Remaining DNA in samples was digested *via* a modified version of the Turbo DNase protocol using the Turbo DNA-free kit (Ambion). The samples were determined to be DNA-free *via* the absence of a band in the agarose gel after PCR amplification (initial denaturation at 95°C for 5 min, denaturation at 95°C for 45 s, annealing at 50°C for 45 s, and elongation at 72°C for 30 s; then repeat steps 2 through 4 for 30 cycles; and final elongation at 72°C for 10 min) using ^519^F/^785^R 16S rRNA primers (^519^F-CAG-CMG-CCG-CGG-TAA and ^785^R-TAC-NVG-GGT-ATC-TAA-TCC) with Escherichia coli K-12 MG1655 DNA as the positive control. The samples were quantified using the Qubit RNA HS assay kit (Invitrogen) and sent to the Department of Energy Joint Genome Institute for rRNA reduction and sequencing using an Illumina NovaSeq S4 2 × 151-nucleotide indexed run protocol (~15 million, 150-bp paired-end reads/sample).

The reads were filtered using BBDuk (v38.92) (9) to remove (i) contaminants, (ii) adapters, (iii) homopolymers of Gs of size 5 or more, (iv) right read segments where quality was 0, (v) reads with N bases, and (vi) reads with an average quality score less than 10, or minimum length of ≤51 bp or 33% of the full length. BBMap (v38.86) (9) removed contaminants (93% identity) and rRNA. Filtered reads were assembled using MEGAHIT (version 1.2.9) (–k-list 23, 43, 63, 83, 103, and 123) (10). The reads were then mapped to assembled contigs using BBMap (ambiguous = random). The assemblies were annotated using the IMG Annotation Pipeline (version 5.0.25) (11).

Taxonomic annotation of protein coding sequences (CDS) by IMG confirmed diatoms were a transcriptionally active component of the winter microbiome in this Laurentian Great Lake. CDS genes annotated as classes Coscinodiscophyceae (centric) and Bacillariophyceae (raphid, pennate) were highly represented relative to other photosynthetic eukaryotes (Table 1). Considering that winters of 2019 (near-maximum ice) and 2020 (negligible ice) spanned extremes in ice cover for Lake Erie, these metatranscriptomes offer a unique opportunity to investigate the influence of climate change on freshwater winter communities.

**TABLE 1:** Assembly and annotation statistics for the 28 winter metatranscriptome samples

Sample ID	Date sampled	Latitude	Longitude	Ice cover (%)	No. of filtered reads	No. of filtered bases	No. of mapped reads	*N*_50_ (bp)	No. of contigs	GC content (%)	No. of CDS genes	CDS genes (%)	CDS genes with product name (%)	Diatom CDS genes (%)	IMG taxon no.
LE1	26 February 2019	41.839333	−82.5215	100	11,544,548	1,665,668,140	8,377,032	16,759	52,540	43.8	56,895	97.6	43.9	48.3	3300048991
LE2	26 February 2019	41.806	−82.383833	100	7,083,156	1,011,185,983	4,982,885	9,235	27,664	43.9	29,724	97.7	46.7	30.8	3300048992
LE3	11 March 2019	41.7497	−81.8352	100	10,264,908	1,470,363,289	7,565,030	16,509	51,873	43.7	57,115	98.8	41.6	26.8	3300048940
LE6	11 March 2019	41.8658	−82.6407	90	6,128,706	869,504,957	3,646,361	9,346	28,093	45.8	30,293	97.1	47.7	40.6	3300048941
LE40	2 March 2020	41.6659	−82.0791	0	6,888,818	995,709,695	4,064,136	14,199	45,111	45.4	51,475	97.7	54.8	33.7	3300048958
LE41 + 47	2 March 2020	41.6659	−82.0791	0	9,116,494	1,312,285,329	5,687,925	21,185	65,087	47.2	73,447	97.9	57.1	31.6	3300048962
LE42 + 46	2 March 2020	41.6659	−82.3516	0	7,883,720	1,133,508,951	6,467,146	6,731	22,608	43.6	23,823	97.2	35.5	49.1	3300048961
LE43	2 March 2020	41.7703	−82.3516	0	9,286,770	1,345,983,724	5,897,712	21,705	68,999	44.4	78,975	98.6	53.9	26.0	3300048959
LE44	2 March 2020	41.7703	−82.3516	0	7,421,002	1,069,834,854	6,538,473	4,146	13,794	41.8	14,534	98.8	34.4	4.4	3300048960
LE45 + 50	2 March 2020	41.7703	−82.3516	0	6,743,964	986,547,861	4,087,436	18,895	58,406	46.4	65,874	98.6	56.5	20.6	3300048964
LE48	2 March 2020	41.7703	−82.3516	0	8,920,712	1,297,708,131	8,159,431	5,752	21,041	41.3	22,118	99.0	36.7	5.6	3300048963
LE49 + 57	14 February 2020	41.8285	−82.5011	0	9,012,522	1,324,397,747	6,390,378	19,319	61,684	48.1	69,326	98.1	52.8	57.6	3300048969
LE52	14 February 2020	41.8285	−82.5011	0	15,967,806	2,351,814,427	11,871,685	32,332	106,157	48.1	121,475	98.6	51.2	60.0	3300048965
LE53	14 February 2020	41.7385	−82.2743	0	9,658,812	1,417,270,672	6,620,172	22,239	72,111	45.8	81,717	98.7	55.1	32.7	3300048966
LE54	14 February 2020	41.6662	−82.0816	0	7,508,292	1,102,118,124	5,023,507	16,765	53,968	48.1	60,671	98.3	53.2	26.7	3300048967
LE56	14 February 2020	41.6662	−82.0816	0	10,823,236	1,585,334,865	7,226,400	24,728	81,868	45.7	92,059	98.6	51.5	21.7	3300048968
LE58	14 February 2020	41.7385	−82.2743	0	7,836,758	1,148,311,846	5,285,429	18,629	60,482	45.0	68,225	98.8	53.2	34.8	3300048970
LE59	14 February 2020	41.6662	−82.0816	0	9,799,816	1,430,143,749	8,060,453	5,511	23,390	40.7	24,515	98.4	38.3	14.0	3300048971
LE60	14 February 2020	41.6662	−82.0816	0	8,010,448	1,172,858,003	7,226,297	5,264	21,397	41.0	22,527	98.6	38.0	15.3	3300048972
LE61 + 65	14 February 2020	41.8285	−82.5011	0	13,876,178	2,045,695,603	12,471,487	12,040	45,535	42.8	48,806	98.7	36.4	53.3	3300049106
LE62	14 February 2020	41.6662	−82.0816	0	9,406,178	1,382,568,183	8,449,070	7,519	33,460	40.3	34,873	98.9	37.3	21.8	3300048973
LE63	14 February 2020	41.7385	−82.2743	0	8,736,118	1,285,818,722	7,914,188	7,339	32,409	39.8	33,149	99.4	35.8	23.2	3300048974
LE64	14 February 2020	41.6662	−82.0816	0	8,906,056	1,311,732,578	7,926,670	8,023	34,284	40.5	35,762	99.0	36.8	26.8	3300049105
LE66	14 February 2020	41.7385	−82.2743	0	6,699,384	985,455,873	6,019,494	6,110	26,186	40.1	27,360	99.4	36.2	20.2	3300049107
LE67 + 70	14 February 2020	41.8285	−82.5011	0	7,502,120	1,107,179,249	6,648,420	9,915	35,134	42.8	37,594	98.8	35.9	55.2	3300049110
LE68	14 February 2020	41.6662	−82.0816	0	8,417,246	1,235,833,865	7,515,913	7,368	32,470	40.4	33,697	99.0	37.6	20.9	3300049108
LE69	14 February 2020	41.7385	−82.2743	0	9,025,914	1,331,601,973	8,118,339	7,553	34,125	39.5	34,728	99.3	34.6	24.1	3300049109
LE71	14 February 2020	41.8285	−82.5011	0	11,197,796	1,647,924,983	10,027,991	11,410	41,266	43.0	43,996	98.7	35.9	56.1	3300049111

Phylogenetic distribution of genes determined by best BLAST hit of coding sequence (CDS) genes at ≥60% identity. The percentage of diatom CDS genes within the eukaryotic CDS genes is reported. Sample IDs consisting of two numbers are indicative of the pooling of the biological replicates.

### Data availability.

Sequences are available through the JGI Genomes Online Database (GOLD) under GOLD Study ID Gs0142002. Assembly and annotation statistics are presented in Table 1. Environmental metadata are available at the Biological and Chemical Oceanography Data Management Office (BCO-DMO).
